# Association of cholesteryl ester transfer protein (*CETP*) gene polymorphism, high density lipoprotein cholesterol and risk of coronary artery disease: a meta-analysis using a Mendelian randomization approach

**DOI:** 10.1186/s12881-014-0118-1

**Published:** 2014-10-23

**Authors:** Zhijun Wu, Yuqing Lou, Xiaochun Qiu, Yan Liu, Lin Lu, Qiujing Chen, Wei Jin

**Affiliations:** Department of Cardiology, Ruijin Hospital, Shanghai Jiao Tong University School of Medicine, Shanghai, 200025 People’s Republic of China; Department of Pulmonary, Shanghai Chest Hospital, Shanghai Jiao Tong University, Shanghai, 200030 People’s Republic of China; Library of Shanghai Jiao Tong University School of Medicine, Shanghai, 200025 People’s Republic of China

**Keywords:** Coronary artery disease, High density lipoprotein cholesterol, Polymorphism, Mendelian randomization

## Abstract

**Background:**

Recent randomized controlled trials have challenged the concept that increased high density lipoprotein cholesterol (HDL-C) levels are associated with coronary artery disease (CAD) risk reduction. The causal role of HDL-C in the development of atherosclerosis remains unclear. To increase precision and to minimize residual confounding, we exploited the cholesteryl ester transfer protein (*CETP*)*-*TaqIB polymorphism as an instrument based on Mendelian randomization.

**Methods:**

The Mendelian randomization analysis was performed by two steps. First, we conducted a meta-analysis of 47 studies, including 23,928 cases and 27,068 controls, to quantify the relationship between the TaqIB polymorphism and the CAD risk. Next, the association between the TaqIB polymorphism and HDL-C was assessed among 5,929 Caucasians. We further employed Mendelian randomization to evaluate the causal effect of HDL-C on CAD based on the findings from the meta-analysis.

**Results:**

The overall comparison of the B2 allele with the B1 allele yielded a significant risk reduction of CAD (P < 0.0001; OR = 0.88; 95% CI: 0.84–0.92) with substantial between-study heterogeneity (I^2^ = 55.2%; P_heterogeneity_ <0.0001). The result was not materially changed after excluding the Hardy-Weinberg Equilibrium (HWE)-violation studies. Compared with B1B1 homozygotes, Caucasian carriers of the B2 allele had a 0.25 mmol/L increase in HDL-C level (95% CI: 0.20–0.31; P <0.0001; I^2^ = 0; P_heterogeneity_ =0.87). However, a 1 standard deviation (SD) elevation in HDL-C levels due to the TaqIB polymorphism, was marginal associated with CAD risk (OR =0.79; 95% CI: 0.54–1.03; P =0.08).

**Conclusions:**

Taken together, our results lend support to the concept that increased HDL-C cannot be translated into a reduction in CAD risk.

**Electronic supplementary material:**

The online version of this article (doi:10.1186/s12881-014-0118-1) contains supplementary material, which is available to authorized users.

## Background

Several clinical parameters are associated with common diseases and are helpful for predicting and preventing these common diseases. For instance, lipid profiles are well acknowledged to be associated with the risk of coronary artery disease (CAD) and myocardial infarction (MI) [1]. Observational and experimental studies have documented a strong positive association of low density lipoprotein cholesterol (LDL-C) and an inverse association of high density lipoprotein cholesterol (HDL-C) with the risk of CAD [2]. Nevertheless, the causal role of HDL-C in the development of atherosclerosis has not been fully clarified and the evidence available from conventional population studies is scarce and paradoxical [3-6]. Many large epidemiological studies, such as the Framingham study [7], have described an increase in HDL-C as being protective to CAD. However, it has been demonstrated that Dalcetrapib and Torcetrapib, two cholesteryl ester transfer protein (CETP) inhibitiors, were shown to elevate circulating HDL-C concentrations substantially, but do not benefit CAD patients in two large randomized controlled trials [8,9]. Both environmental exposures and genetic factors are thought to contribute to the majority of these inconsistencies. Deciphering the regulation of HDL-C metabolism via the interaction of inherited variations with environmental factors may help explore the underlying pathological mechanism of CAD.

CETP plays a key role in determining the circulating HDL levels and transfers cholesteryl esters from HDL-C to LDL-C, as well as very low density lipoprotein cholesterol (VLDL-C), in exchange for triglyceride rich lipoproteins [10]. An inverse association of CETP activity with HDL-C levels was observed [11]. Some genome-wide association studies have suggested that the correlation of the *CETP* locus with HDL-C concentrations is more significant than that of any other locus found across the genome [12,13]. Among these genetic variants, the TaqIB polymorphism (rs708272) in intron 1 [14] has been investigated extensively [15]. Significant associations of the TaqIB polymorphism with variations in CETP promoter activity [16] and circulating HDL-C concentrations [14] were observed, showing lower CETP concentrations and higher HDL-C levels among B2 carriers than among B1B1 homozygotes [14,17-19]. However, the definite relationship between the TaqIB polymorphism and CAD encountered a considerable dispute among the genotype-disease studies [12,19-23], most likely due to relatively small sample sizes, limitations in statistical power and interactions with ethnic descents, population classifications and environment exposures. We hypothesized that a large meta-analysis is a robust method that can reliably appraise the heterogeneity present in genetic association studies and also can expound the divergences. Given that the HDL-C is an intermediate in the causal pathway from the *CETP* gene to CAD, it would be sensible to conduct a meta-analysis that in some way integrates the triangle relationship: the TaqIB polymorphism-HDL-C (genotype-phenotype), the TaqIB polymorphism-CAD (genotype-disease), and HDL-C-CAD (phenotype-disease). The logic of this approach is greatly strengthened by the appeal to Mendelian randomization [24], which is according to Mendel’s second law (the law of independent assortment) [25]. Mendelian randomization means that the inheritance of an individual’s genes is independent by a seemingly random process at conception. Theoretically, the genetic polymorphism, which is causally and specifically bound up with the intermediate phenotype and the predicted risk of disease, could be exploited as an instrument to attain an un-confounded estimation of phenotype-disease association. In the present study, we examined whether the increase in circulating HDL-C concentrations due to the TaqIB polymorphism was correlated with a reduced risk of CAD, based on the rationale of Mendelian randomization [26].

## Methods

### Literature search strategy

Two authors (Wu and Lou) independently performed the search. The process was supervised by the third author (Qiu). Any disagreement was resolved by a consensus. The advanced search builder was used to refine the search. The Boolean operators “AND” and “OR” were used to combine the search themes. A stepwise search was conducted as recommended by the Cochrane Handbook for Systematic Reviews of Interventions (Version 5.1.0). A formal computerized literature search of electronic databases, PubMed/MEDLINE, Embase, CNKI (China Nation Knowledge Infrastructure Platform), Wanfang and CBM (China Biological Medicine), was conducted up to March 2014. A composition of the following MeSH terms and text words was used: ‘TaqIB’ or ‘rs708272’, ‘cholesteryl ester transfer protein’ or ‘CETP’, ‘high density lipoprotein cholesterol’ or ‘HDL-C’ and ‘coronary artery disease’ or ‘myocardial infarction’ or ‘atherosclerosis’. The additional studies were retrieved from the MEDLINE option ‘related articles’ and head searches were also added to the database. We also screened the bibliographies of the original research reports, reviews, and previous meta-analyses to optimize the databank. If the data was deficient or in an inappropriate form, we contacted with the original authors to obtain the raw data.

### Selection criteria

All the studies that aimed for the association of the TaqIB polymorphism with HDL-C levels and CAD risk were potentially included. The following criteria were for the selection: (1) published articles of human-being genetics (full texts or abstracts) without racial or language restrictions; (2) if articles contained more than one geographic or other clinical characteristic subgroup, each subgroup was considered separately; (3) if multiple studies were derived from the same population, only the study with the largest sample size was involved to avoid overlapping data; (4) studies providing sufficient information on the TaqIB genotype by case–control status and/or on circulating HDL-C concentrations across the TaqIB genotype between CAD patients and controls.

CAD outcomes were diagnosed according to previous MI, angina pectoris, percutaneous trans luminal coronary angiography, coronary artery bypass grafting or severe angiographic stenosis ( ≥50% of ≥1 major coronary artery) [27]. Acute coronary syndrome (ACS) included unstable angina pectoris, as well as fatal and non-fatal MI [28]. MI was defined by the World Health Organization’s Multinational Monitoring of Trends and Determinants in Cardiovascular Disease (MONICA) criteria [29].

### Extracted information

A standard data-collection procedure, in line with the inclusion criteria described above was used. Two investigators (Wu and Lou) independently extracted the variables from the individual eligible studies in duplicate and made the characteristics compatible in a pooled database. Any encountered disagreements were resolved by discussion in order to reach a consensus. The following information was extracted from all the eligible studies: first author’s name, publication year, ethnicity, geographic location, study design, population source, endpoints, clinical characteristics of the study subjects (such as age, gender, body mass index [BMI] and circulating lipid profiles levels), percentage of hypertension, diabetes, smoking status and the distribution of the TaqIB genotype both in patients and controls. The units of circulating lipid profiles were standardized to mmol/L. Continuous variables were expressed as mean ± standard deviation (SD) or median (5th and 95th percentiles). Standard error was converted to SD.

### Hardy-Weinberg Equilibrium (HWE) testing

We tested conformity of the TaqIB polymorphism to HWE among controls in each individual study via the chi-square test or Fisher’s exact test, based on a Web program (http://ihg2.helmholtz-muenchen.de/cgi-bin/hw/hwa1.pl). In order to obtain robust evidence of estimating the association between the TaqIB polymorphism and CAD risk, sensitivity analyses were performed by excluding the HWE-deviating studies (P <0.05) [30]. We calculated the effect sizes for all the studies and then we calculated the effect sizes only for HWE-conforming studies.

### Statistical analysis

Initially, we calculated the summary odd ratios (ORs) of CAD risk and the standard mean difference (SMD) of HDL-C concentrations corresponding to the 95% confidence interval (CI) to assess whether the TaqIB polymorphism was relevant to CAD risk or circulating HDL-C levels or both. Four genetic models, including allele comparison (B2 versus B1), dominant genetic model (B1B2 + B2B2 versus B1B1), recessive genetic model (B2B2 versus B1B2 + B1B1) and homozygote comparison (B2B2 versus B1B1) were used. We utilized a random-effects model based on the DerSimonian & Laird method to evaluate the effect size of each study and to modify the study weights on the basis of the in-study variance. The Mantel-Haenszel model was adopted for checking the possibility of heterogeneity [31]. Uniformity of findings across all studies was estimated by means of the inconsistency index (I^2^) statistic, ranging from 0 to 100%. A value of 0% indicated homogeneity; by contrast, high values of I^2^ implied that heterogeneity accounted for most of the between-study variation [32,33]. The between-study heterogeneity was differentiated with a Chi-square-based Q statistic test [34] where P <0.1 indicated heterogeneity across the studies. The significance level of the combined ORs which was estimated by the Z test, was P <0.05. Next, we examined pre-specified groupings of study characteristics with homogeneous effects, such as ethnicity (Asian, Caucasian and mixed-population), study design (prospective and retrospective), population source (hospital-based [H-B] and population-based [P-B]), and endpoints (CAD, MI and ACS). In addition, we conducted a meta-regression, as a complement to the estimation of the relationship between the TaqIB polymorphism and CAD risk to find the potential effect of environmental covariates on genetic heterogeneity.

Sensitivity analysis was performed by sequentially removing each individual study to identify those that likely biased the overall estimates. We used the visual funnel plot and Egger’s linear regression test to estimate publication bias. The standard error of log (OR) for each study was plotted against its OR. An asymmetric plot suggests the possible presence of publication bias, which can be verified by a T-test. P <0.05 of the I^2^ statistic and Egger’s test was regarded as significant [35].

Cumulative meta-analysis was used to decipher the impact of the first published study on the subsequent studies and the evolution of the synthesized effects over time was in accordance with the publications ordered by time. Data administration and statistical analysis were conducted by using Review Manager software release 5.0 (Oxford, UK) and Stata 11.0 (Stata Corporation, College Station, TX, USA). All P values were 2-sided.

## Results

### Results of study search and characteristics

**Figure 1:**
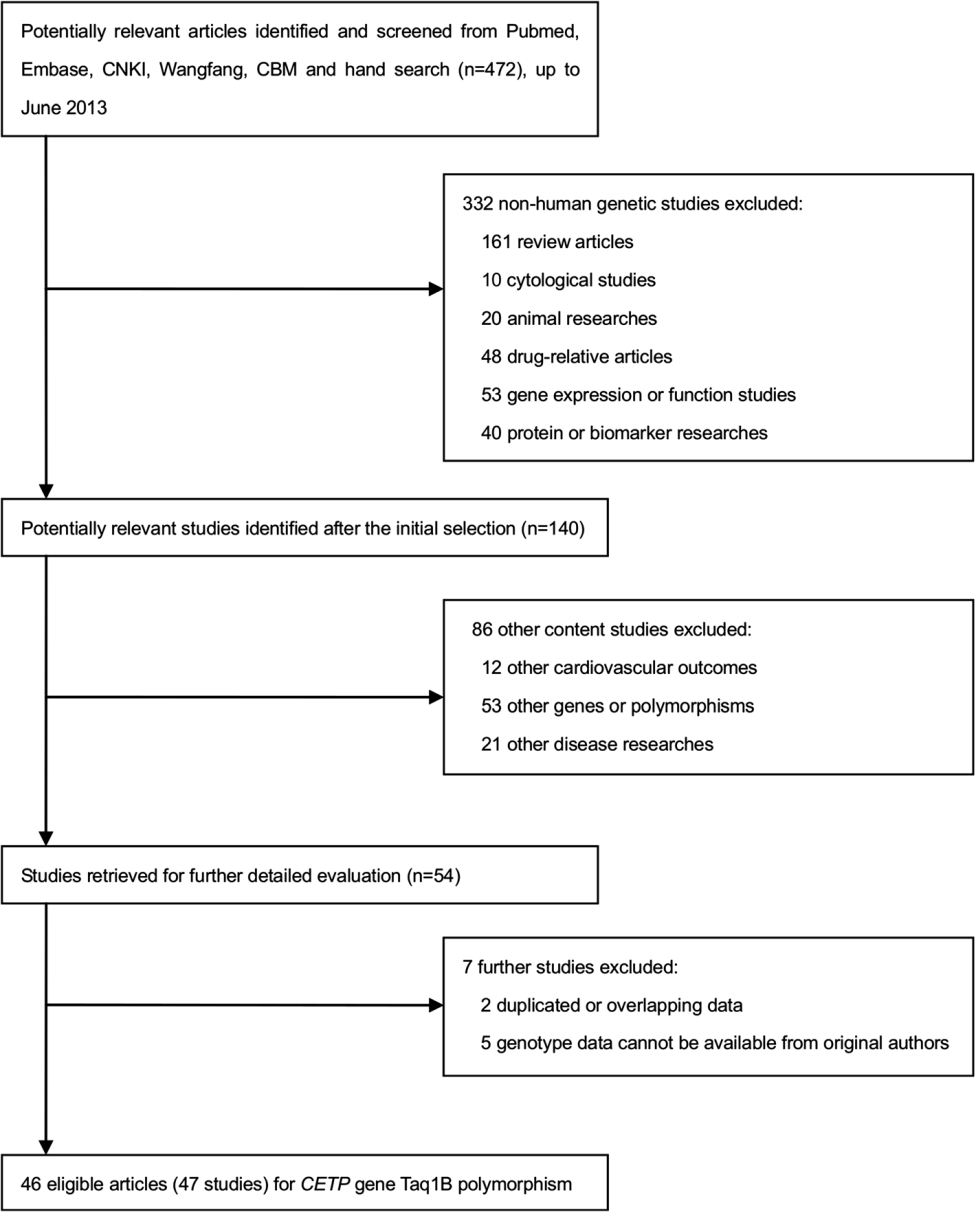
**Flow diagram of the search strategy and study selection for the meta-analysis.**

The flowchart depicting the screening process of study selection is shown in Figure 1 and the excluded articles, with the reasons for exclusion are also described. A total of 470 relevant publications were obtained by the preliminary search in PubMed, EMBASE, CNKI, Wangfang and CBM. After evaluation for our inclusion criteria, 46 articles including 47 studies with adequate information related to the association between the TaqIB polymorphism and CAD risk, were included in the final analysis [11,17,20-22,36-66]. The clinical characteristics of the eligible studies are summarized in Additional file 1: Table S1. All the eligible studies were published between 1991 and 2012.There were 20 studies performed in Asian subjects [11,38,47,51,57,58,60,63,65,66], 26 in Caucasian subjects [17,20-22,36,37,40-46,49,50,52-56,59,61,62,64,67] and one mixed- population study [48]. In consideration of the study design, 6 studies were prospective [20,40,43,45,49] and the remaining 41 studies were retrospective [11,17,21,22,36-38,41,42,44,46-48,50-67]. In addition,11 of the 47 studies were P-B [20-22,36,37,40,43,46,49,54] and the remaining studies were H-B [11,17,38,39,41,42,44,45,47,48,50-53,55-66]. CAD was regarded as the main outcome in most of the studies [11,17,21,22,36-40,42,44,45,47,49-51,53,55-61,64-66], except 8 studies analyzed MI [20,43,46,48,52,54,62,63] and 2 studies analyzed ACS [41] as the endpoint. A significant departure from HWE was found in 7 studies [21,39,48,52,63]. The article by Jensen et al. [49] contained data from two independent studies (Nurses’ Health Study [NHS] and Health Professionals Follow-up Study [HPFS]); therefore we calculated the ORs separately in our meta-analysis. The study descriptions were shown in Additional file 2.

### Overall analysis

Forty-seven studies with 23,928 cases and 27,068 controls were incorporated into the meta-analysis. The allele and genotype frequency of the TaqIB polymorphism is shown in Table 1. The results of HWE examination among the controls for all the eligible studies are also listed. The synthetic overall percentage of the B2 allele was 41.4% in cases and 43.7% in controls. The B2 allele had a similar proportion among Asians subjects (40.1% in cases versus 47.1% in controls) compared to that among Caucasian subjects (41.2% in cases versus 42.5% in controls).

**Table 1:** **The distribution of the Taq1B allele and genotype among CAD and controls, and P value of HWE in controls**

	**Sample size**		**B1 allele, %**		**B2 allele, %**		**B1B1 genotype**		**B1B2 genotype**		**B2B2 genotype**		**HWE,**
**First author**	**Cases**	**Controls**	**Cases**	**Controls**	**Cases**	**Controls**	**Cases**	**Controls**	**Cases**	**Controls**	**Cases**	**Controls**	**P value**
Arca M	415	403	60.4	58.1	39.6	41.9	153	134	187	184	68	71	0.57
Bhanushali AA	90	150	59.4	50.3	40.6	49.7	33	37	40	76	17	42	0.820
Blankenberg S	1214	574	60.8	57.2	39.3	42.8	407	175	644	303	149	93	0.047
Corella D	557	1180	62.4	63.6	37.6	36.4	224	482	247	537	86	161	0.557
Dedoussis GV	237	237	60.5	58.2	39.5	41.8	83	78	121	120	33	39	0.530
Durlach A	96	138	-^a^	-	-	-	-	-	-	-	13	39	-
Eiriksdottir G	388	794	59.1	52.6	40.9	47.4	128	194	191	396	59	155	0.072
Falchi A	100	100	58.5	56.0	41.5	44.0	30	30	57	52	13	18	0.581
Freeman DJ	498	1108	58.8	55.2	41.2	44.8	164	339	259	541	76	225	0.733
Fumeron F	608	724	60.0	59.5	40.0	40.5	209	258	312	346	87	120	0.826
Horne BD	3223	1588	57.5	56.0	42.5	44.0	1064	508	1579	762	580	318	0.293
Hsieh MC	101	264	42.1	29.7	57.9	70.3	19	23	47	111	35	130	0.920
Izar MC	386	604	40.1	43.0	59.9	57.0	32	66	238	374	107	145	0.000
Jensen MK [HPFS]	259	513	58.7	58.9	41.3	41.1	89	180	126	244	44	89	0.686
Jensen MK [NHS]	246	486	58.5	58.3	41.5	41.7	84	166	120	235	42	85	0.907
Kaestner S	204	35	53.9	60.0	46.1	40.0	53	13	114	16	37	6	0.778
Kawasaki I	24	361	79.2	53.6	20.8	46.4	15	101	8	185	1	75	0.565
Keavney B	4685	3460	57.7	56.9	42.3	43.1	1477	1100	2175	1527	790	646	0.005
Kolovou G	374	97	60.7	46.4	39.3	53.6	126	22	202	45	46	29	0.573
Li J	236	54	58.8	58.8	41.2	41.3	82	15	73	19	21	6	0.997
Liu S	384	384	58.1	56.9	41.9	43.1	125	122	196	193	63	69	0.628
McCaskie PA	556	2683	59.3	57.0	40.7	43.0	196	860	262	1328	93	485	0.482
Meiner V	577	659	57.1	52.6	42.9	47.4	173	166	282	320	95	134	0.383
Mohrschladt MF	116	184	56.9	54.9	43.1	45.1	36	57	60	88	20	39	0.642
Muendlein A	332	225	62.0	57.3	38.0	42.7	125	71	162	116	45	38	0.420
Padmaja N	504	338	58.5	49.3	41.5	50.7	163	86	264	161	77	91	0.386
Park KW	119	106	-	-	-	-	49	30	-	-	-	-	-
Poduri A	265	150	64.3	49.3	35.7	50.7	117	33	107	82	41	35	0.252
Porchay-Balderelli I	223	2901	63.9	59.5	36.1	40.5	95	1012	95	1431	33	458	0.198
Qin Q	249	167	58.8	58.4	41.2	41.6	81	49	131	97	37	21	0.012
Rahimi Z	207	92	62.3	50.0	37.7	50.0	57	20	144	52	6	20	0.211
Rejeb J	212	104	71.0	65.9	29.0	34.1	104	45	93	47	15	12	0.959
Schierer A	349	2082	47.0	49.0	53.0	51.0	-	-	-	-	-	-	-
Tenkanen H	72	226	54.2	55.5	45.8	44.5	19	64	40	123	13	39	0.125
Van Acker BA	792	539	59.4	58.1	40.6	41.9	275	171	391	284	126	84	0.06
Wang SH	111	75	58.6	56.0	41.4	44.0	38	22	54	41	19	12	0.327
Wang W	128	247	64.8	54.0	35.2	46.0	50	72	66	123	12	52	0.968
Whiting BM	3319	1385	58.1	56.7	41.9	43.3	792	280	1201	377	402	170	0.039
Wu JH	200	285	56.7	52.0	43.3	48.0	45	63	79	159	25	52	0.007
Yan SK	106	64	60.4	56.3	39.6	43.8	41	19	46	34	19	11	0.526
Yang J	83	163	62.7	52.1	37.3	47.9	31	47	42	76	10	40	0.401
Yilmaz H	173	111	59.0	55.5	41.0	44.5	66	39	72	46	35	26	0.093
Zhang GB	88	94	58.0	60.6	42.0	39.4	31	32	40	50	17	12	0.268
Zhang YX	334	301	67.7	65.1	32.3	34.9	174	136	104	120	56	45	0.034
Zhao SP	238	203	62.0	56.4	38.0	43.6	95	60	105	109	38	34	0.191
Zheng KQ	203	100	60.6	60.5	39.4	39.5	66	33	114	55	23	12	0.132
Zhou DF	47	330	57.4	68.0	42.6	32.0	17	157	20	135	10	38	0.280
Total	23928	27068	58.6	56.3	41.4	43.7	7533	7667	10910	11720	3634	4521	

HWE: Hardy-Weinberg equilibrium. The P value of HWE determined by the χ2 test or Fisher’s exact test in control groups; a: No data.

**Figure 2:**
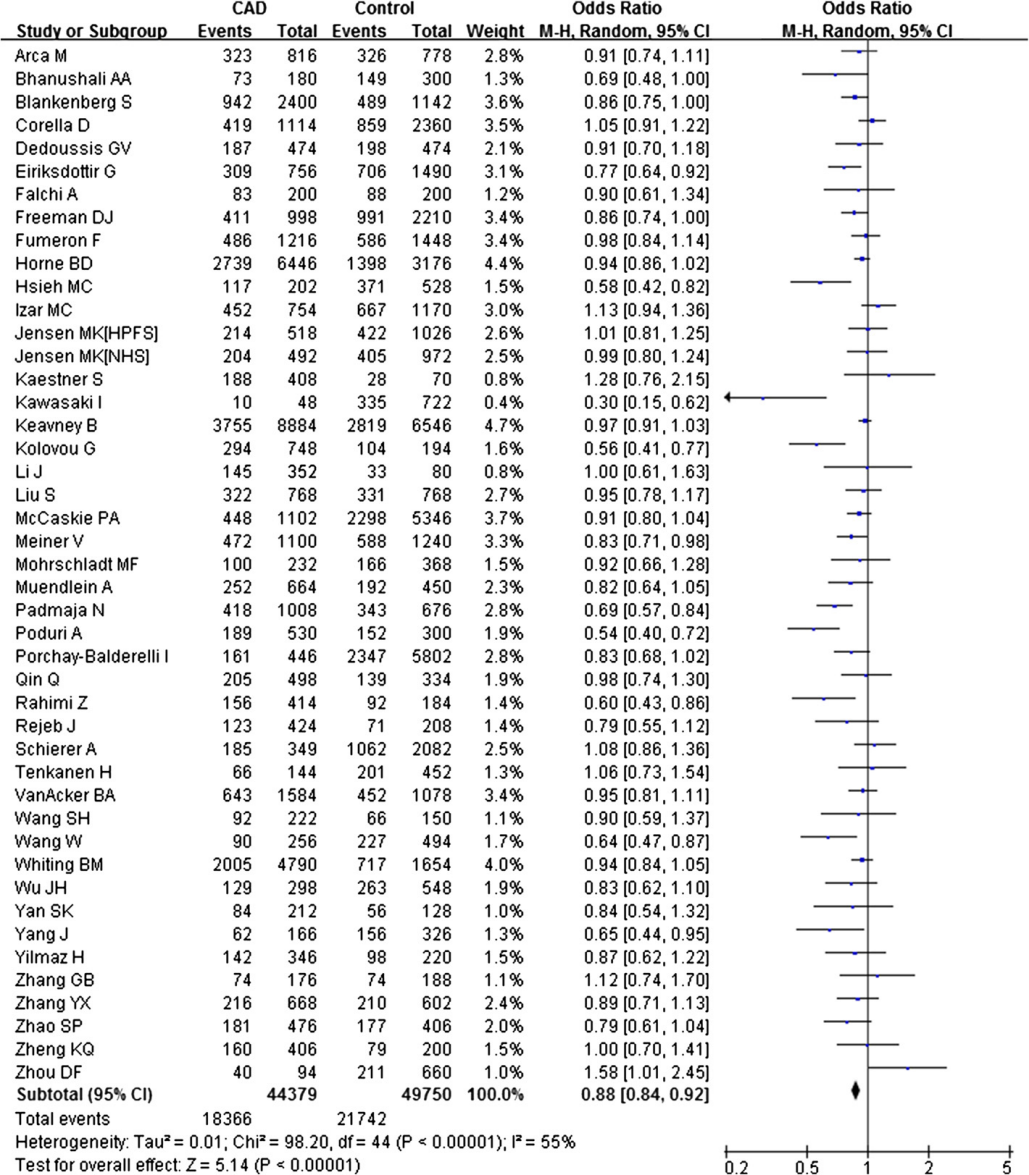
**Meta-analysis for the overall association between the** ***CETP*** **TaqIB polymorphism and CAD under the allele comparison (B2 versus B1).** ‘Events’ indicates the total number of the B2 allele. ‘Total’ indicates the total number of B2 allele plus B1 allele.

We assessed the association between the TaqIB polymorphism and CAD risk for each study under different genetic models (Table 2).The overall comparison of the B2 allele with the B1 allele demonstrated a significant risk reduction of CAD (allele comparison: P <0.0001, OR = 0.88, 95% CI: 0.84–0.92; dominant model: P <0.0001, OR = 0.85, 95% CI: 0.79–0.91) with substantial between-study heterogeneity (allele comparison: I^2^ = 55.2%, P_heterogeneity_ <0.0001; dominant model: I^2^ = 48.6%, P_heterogeneity_ <0.0001) (Figure 2).

**Table 2:** **Summary estimates for ORs and 95% CI in different subgroups under various genetic contrasts**

**Genotype contrasts**	**Study population**	**Study number, (case/control), n(n/n)**	**P**_**heterogeneity**_	**I** ^**2**^ **, %**	**P value** ^**a**^	**OR**	**95% CI**
Total studies							
Allele comparison		45(23,713/26,824)	0.000	55.2	0.000	0.88	0.84–0.92
(B2 versus B1)							
Dominant model		45(23,483/24,848)	0.000	48.6	0.000	0.85	0.79–0.91
(B1B2 + B2B2 versus B1B1)							
Recessive model		45(23,460/24,880)	0.000	53.7	0.000	0.81	0.74–0.88
(B2B2 versus B1B2 + B1B1)							
Homozygote comparison		44(23,364/24,742)	0.000	58.1	0.000	0.76	0.68–0.84
(B2B2 versus B1B1)							
Studies comfirming to HWE							
Allele comparison		38(13,326/20,048)	0.000	56.6	0.000	0.86	0.81–0.91
Dominant model		38(13,096/18,072)	0.002	45.1	0.000	0.82	0.76–0.89
Recessive model		38(13,073/18,104)	0.000	55.1	0.000	0.77	0.69–0.86
Homozygote comparison		37(12,977/17,966)	0.000	59.2	0.000	0.72	0.63–0.82
Subgroups analysis after excluding HWE-deviation studies							
*Ethnicity*							
Allele comparison	Asian	16(2,780/4,767)	0.000	67.7	0.002	0.77	0.66–0.90
	Caucasian	22(10,546/15,281)	0.222	18.0	0.000	0.91	0.87–0.95
Dominant model	Asian	16(2,550/2,791)	0.022	46.3	0.000	0.65	0.54–0.79
	Caucasian	22(10,546/15,281)	0.505	0	0.001	0.90	0.85–0.96
Recessive model	Asian	15(2,431/2,685)	0.000	66.7	0.008	0.66	0.49–0.90
	Caucasian	23(10,642/15,419)	0.106	27.9	0.000	0.84	0.76–0.92
Homozygote comparison	Asian	15(2,431/2,685)	0.000	66.6	0.000	0.54	0.38–0.76
	Caucasian	22(10,546/15,281)	0.159	23.2	0.000	0.82	0.74–0.90
*Study design*							
Allele comparison	prospective	7(2,555/7,366)	0.116	41.3	0.061	0.92	0.84–1.00
	retrospective	31(10,771/12,682)	0.000	59.2	0.000	0.84	0.78–0.90
Dominant model	prospective	7(2,555/7,366)	0.161	35	0.081	0.89	0.79–1.01
	retrospective	31(10,518/10,738)	0.002	47.3	0.000	0.79	0.72–0.88
Recessive model	prospective	7(2,555/7,366)	0.219	27.4	0.119	0.89	0.76–1.03
	retrospective	31(10,518/10,738)	0.000	58.1	0.000	0.73	0.64–0.84
Homozygote comparison	prospective	7(2,555/7,366)	0.106	42.7	0.080	0.84	0.70–1.02
	retrospective	30(10,422/10,600)	0.000	61.6	0.000	0.67	0.57–0.79
*Population source*							
Allele comparison	P-B	10(4,782/8,365)	0.308	14.7	0.015	0.93	0.88–0.99
	H-B	28(8,544/11,683)	0.000	60.6	0.000	0.82	0.75–0.89
Dominant model	P-B	10(4,782/8,365)	0.361	8.8	0.025	0.91	0.84–0.99
	H-B	28(8,314/9,707)	0.002	49.5	0.000	0.76	0.68–0.86
Recessive model	P-B	10(4,782/8,365)	0.568	0	0.055	0.91	0.82–1.00
	H-B	28(8,291/9,739)	0.000	59.1	0.000	0.70	0.59–0.82
Homozygote comparison	P-B	10(4,782/8,365)	0.292	16.4	0.020	0.86	0.76–0.98
	H-B	27(8,195/9,601)	0.000	62.7	0.000	0.63	0.52–0.77
*Endpoint*							
Allele comparison	CAD	31(10,824/16,970)	0.000	62	0.000	0.85	0.79–0.91
	MI	5(2,029/2,787)	0.200	33.2	0.032	0.89	0.80–0.99
	ACS	2(473/291)	0.741	0	0.517	0.93	0.74–1.17
Dominant model	CAD	31(10,594/14,994)	0.001	49.5	0.000	0.81	0.73–0.89
	MI	5(2,029/2,787)	0.123	44.8	0.172	0.88	0.74–1.06
	ACS	2(473/291)	0.493	0	0.357	0.86	0.61–1.19
Recessive model	CAD	31(10,571/15,026)	0.000	62.8	0.000	0.75	0.66–0.87
	MI	5(2,029/2,787)	0.775	0	0.006	0.80	0.69–0.94
	ACS	2(473/291)	0.905	0	0.356	0.81	0.52–1.27
Homozygote comparison	CAD	30(10,475/14,888)	0.000	65.2	0.000	0.69	0.59–0.82
	MI	5(2,029/2,787)	0.300	17.9	0.008	0.76	0.63–0.93
	ACS	2(473/291)	0.723	0	0.273	0.76	0.46–1.24

^a^Test for overall effect; P-B: population-based, H-B: hospital-based.

### Sensitivity analysis

We hypothesized that HWE-deviating studies (7 studies containing 10,387 cases and 6,776 controls) might be partly responsible for the striking heterogeneity. Therefore, we performed a sensitivity analysis to compare the summary effects as well as the extent of between-study heterogeneity before and after excluding HWE-deviating studies. However, the summary ORs and the statistical significance of tests did not change substantially after removal of the HWE-deviating studies (allele comparison: P <0.0001, OR =0.86, 95% CI: 0.81–0.91, I^2^ = 56.6%, P_heterogeneity_ <0.0001; dominant model: P <0.0001, OR = 0.82, 95% CI: 0.76–0.89, I^2^ = 45.1%, P_heterogeneity_ <0.0001). In addition, no individual study was shown to substantially influence the overall results.

### Publication bias

**Figure 3:**
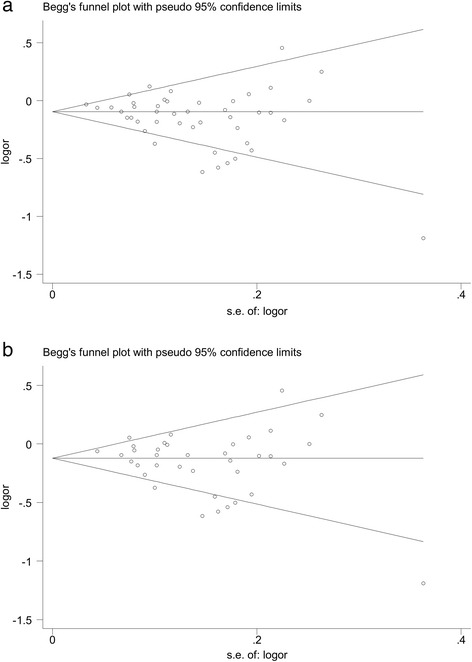
**Begg’s funnel plot analysis to detect publication bias for allele comparison (B2 versus B1) of the TaqIB polymorphism (a) and after excluding the HWE-violating studies (b).**

A remarkable publication bias of all the studies was reflected by the asymmetry of the funnel plot and verified by Egger’s regression test (t = −2.48, P =0.02 for allele comparison), but not verified by the Begg-Mazemdar test (P =0.19 for allele comparison). Nevertheless, after excluding the HWE-violating studies, the probability of publication bias was diminished and the P value for asymmetry in the funnel plot became non-significant (t = −1.71; P =0.10 for Egger’s regression test and P =0.44 for Begg-Mazemdar test). Further analysis via the trim and fill method suggested that no missing studies were needed to adjust the symmetry in the funnel plot for the TaqIB polymorphism (Figure 3).

### Cumulative analysis

No distinguishable evidence was found that the first published study affected the subsequent replication, as illustrated by the cumulative meta-analysis (data not shown).

### Subgroup analysis

In view of the considerable heterogeneity in the total analysis, subgroup analysis is an appropriate method to explore the potential sources of heterogeneity. We categorized the data in the light of the different homogeneous characteristics, such as ethnicity, study design, population source and disease type in HWE-confirming studies. The summary estimates for ORs in different subgroups under various genetic contrasts are listed in Table 2. Population stratification by ethnicity detected that the magnitude of risk reduction, given by the TaqIB polymorphism among Asian subjects, was much greater than that among Caucasian subjects, being 23% (95% CI: 0.66–0.90, P =0.002) and 9% (95% CI: 0.87–0.95, P <0.0001), respectively. Discernible heterogeneity among Asian subjects (I^2^ = 67.7%, P_heterogeneity_ <0.0001) was not observed among Caucasian subjects (I^2^ = 18%, P_heterogeneity_ =0.22). Further, the comparison of the B2 allele with the B1 allele yielded a non-significant 8% risk reduction for CAD in the prospective group (95% CI: 0.84–1.00, P = 0.06, I^2^ = 41.3%, P_heterogeneity_ =0.12), which was higher than that in the retrospective group (OR = 0.84, 95% CI: 0.78–0.90, P <0.0001, I^2^ = 59.2%, P_heterogeneity_ <0.0001). Upon stratification by population source, the ORs of CAD appeared to decrease in the H-B group (OR =0.82, 95% CI: 0.75–0.89, P <0.0001, I^2^ = 60.6%, P_heterogeneity_ <0.0001) relative to the P-B group (OR = 0.93, 95% CI: 0.88–0.99, P =0.02, I^2^ = 14.7%, P_heterogeneity_ =0.308). In addition, the risk estimate was less in the MI and ACS groups than in the CAD group (OR = 0.85, 95% CI:0.79–0.91, P <0.0001, I^2^ = 62%, P_heterogeneity_ <0.0001). Carrier status for the B2 allele carried a moderate risk reduction of MI (OR = 0.89, 95% CI: 0.80–0.99, P =0.03, I^2^ = 33.2%, P_heterogeneity_ = 0.2) and a suggestive risk reduction of ACS (OR =0.93, 95% CI: 0.74–1.17, P =0.52, I^2^ = 0%, P_heterogeneity_ =0.74).

### Meta-regression analysis

**Figure 4:**
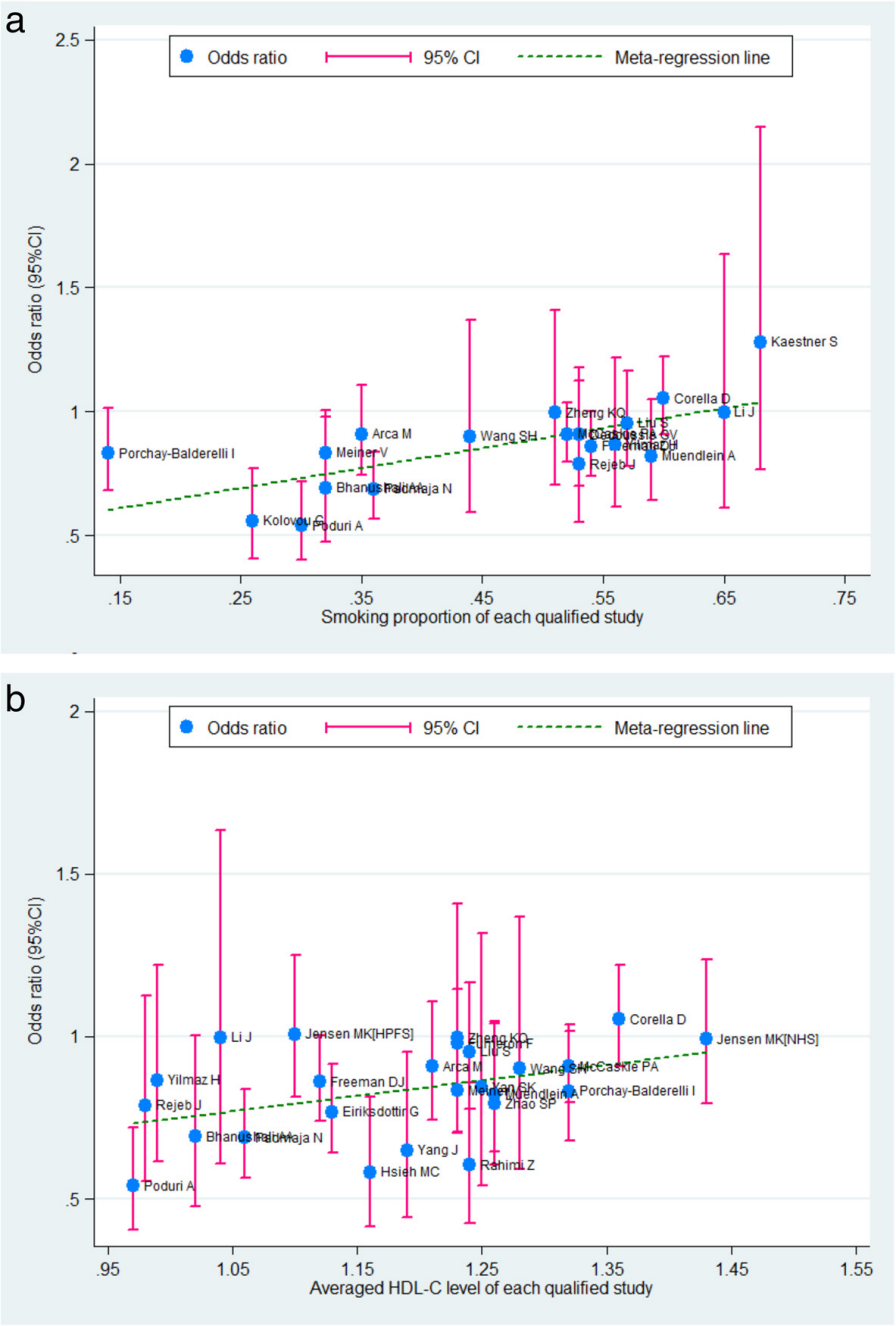
**Meta-regression of overall smoking proportion (a) and averag HDL-C level (b) on in-allele risk estimates of the TaqIB polymorphism.** OR is expressed as the middle of the blue solid circle whose upper and lower extremes represent the corresponding 95% CI. The green dotted line is plotted by fitting OR with overall smoking proportion **(a)** and averaged HDL-C level **(b)** for each included study.

The major environmental exposures were added in a series of univariate models to interpret the potential sources for between-study heterogeneity. Multiple study-level covariates, including average age, BMI, lipid fractions, percentage of male participants, smoking, hypertension and diabetes, were incorporated in our meta-regression. As a result, the CAD risk estimate for the TaqIB polymorphism was significantly influenced by smoking status (P = 0.006) and by circulating HDL-C levels (P = 0.006). This indicates that the correlation of the TaqIB polymorphism with CAD is likely to be strengthened in populations having low smoking rates or low HDL-C levels, with the reduction of CAD risk due to the B2 allele being greater in populations with low smoking rates compared with populations with high smoking rates and in populations with low HDL-C levels compared with populations with high low HDL-C levels (Figure 4).

### Association of genotype-phenotype

**Figure 5:**
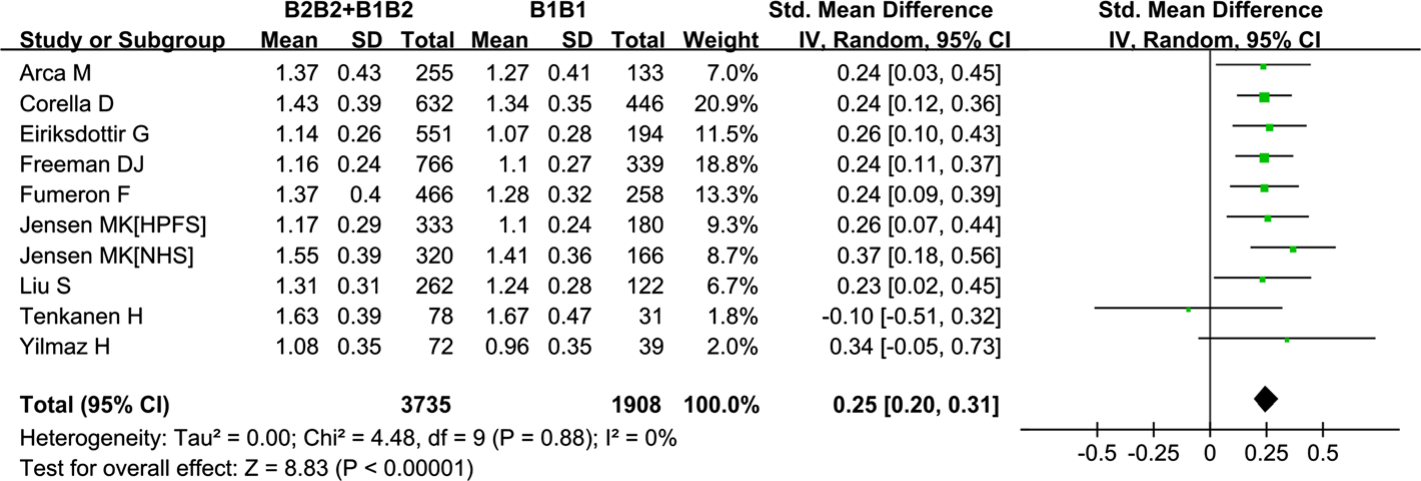
**Meta-analysis for the association between the** ***CETP*** **TaqIB polymorphism and circulating HDL-C level among Caucasians under the dominant model (B2B2 + B1B2 versus B1B1).** ‘SD’ indicates standard deviation. ‘Total’ indicates the number of measured participants.

To assess the specificity of HDL-C, we evaluated the association of the TaqIB polymorphism with HDL-C in 10 studies of Caucasian subjects, which contained 3,600 cases and 5,929 controls. Considering that some drugs such as statins probably influence circulating HDL-C levels and are generally used in treatment of CAD and MI, we calculated the SMD only among the controls. As expected, the HDL-C concentrations were dramatically increased in the B2 carriers without evidence of between-study heterogeneity. Carrier status for the B2 allele was associated with an increase of roughly 0.25 mmol/L in HDL-C (B2B1 + B2B2 versus B1B1: SMD = 0.25, 95% CI: 0.20–0.31, P <0.0001, I^2^ = 0, P_heterogeneity_ = 0.87) (Figure 5) with a low probability of publication bias, as reflected by the Egger’s test (t = 0.69, P = 0.51) and Begg-Mazemdar test (P = 0.37). None of the environmental exposures mentioned above influenced the between-study heterogeneity.

### Predicted relationship of phenotype-disease from Mendelian randomization

According to Mendelian randomization, the strength of the instrument (the TaqIB polymorphism in our study) is determined by the absolute magnitude of its association with the intermediate phenotype (HDL-C in our study). A previous individual patient data-based meta-analysis, including 13,677 Caucasian subjects, provided robust evidence that the TaqIB polymorphism is significantly associated with HDL-C among Caucasians [18]. Hence, we think the TaqIB polymorphism is a qualified predictive instrument when using a Mendelian randomization approach among Caucasians combined with our above genotype-disease results. Given most CAD and MI patients use lipid-lowering drugs, such as statins, as a primary therapy, which had a potential influence on circulating HDL-C levels, we estimated the association between HDL-C and CAD only among the controls. Among the 40 studies conforming to HWE, 14 studies (10 Caucasian studies and 4 Asian studies) provided complete information about both the TaqIB genotype -CAD association and the TaqIB genotype-HDL-C association [20,36,40,43,45,46,49,58,60,62,64,65]. The other 26 studies only estimated the association between the TaqIB genotype and CAD in detail and did not provide adequate information concerning HDL-C.

To test the reliability and robustness of our data, we did a statistical power analysis as previously described [68,69]. We assumed that OR equals 1.5 and 2.0 for differences in allele frequency, the minor allele frequency is 0.38 and a preset threshold value (α) to reject a null hypothesis is 0.05. As a result, while an allele that has an OR of 1.5 and 2, 80% and 100% of the studies achieved 80% statistical power, respectively. The statistical power of the overall comparison achieved 100% (Table 3).

**Table 3:** **Expected power analysis of the TaqIB polymorphism**

**First author**	**Expected power, %** ^**a**^	
	**OR = 1.5**	**OR = 2**
Arca M	98.2	100
Corella D	100	100
Eiriksdottir G	99.6	100
Freeman DJ	100	100
Fumeron F	99.9	100
Jensen MK [HPFS]	96.1	100
Jensen MK [NHS]	95.2	100
Liu S	97.5	100
Tenkanen H	55.8	95.1
Yilmaz H	64.2	98
Total	100	100

OR: Odds ratio. ^a^assuming OR of 1.5 and 2.0 for differences in allele frequency, the minor allele frequency of 0.38 and Type I error probability α of 0.05.

Given the close relationship between the TaqIB polymorphism and HDL-C in these 10 studies of Caucasian subjects, we ascertained that B2 carriers had a decreased risk of CAD by 6% (95% CI: 0.86–1.03, P =0.20, I^2^ = 0, P_heterogeneity_ =0.52). There seems to be no evidence of publication bias (t = −0.11, P =0.92 for Egger’s test). Utilizing the TaqIB polymorphism as an instrument, we measured the magnitude of the association between increased HDL-C concentrations with CAD under the assumptions required for Mendelian randomization. The Mendelian randomization estimate was computed on the basis of assumed linearity of the association between HDL-C variation and OR for CAD on a log scale [70]. Considering that the National Cholesterol Education Program (NCEP) has set the guidelines for lipids and HDL-C <1.04 mmol/L (40 mg/dL) is considered low HDL-C and HDL-C >1.55 mmol/L (60 mg/dL) is high HDL-C [71], we established a concentration gradient based on increased amounts of HDL-C (mmol/L): 0.03, 0.05, 0.10, 0.20, 0.30, 0.50 and 1.00. The results suggest a marginal significant association between genetically increased circulating HDL-C levels and the reduced risk of CAD (OR = 0.79 per 1 mmol/L increase in HDL-C, 95% CI: 0.54–1.03, P =0.08). Although the estimate size remains borderline significant, we observed a tendency showing a greater increase in HDL-C to have a more significant reduction of CAD risk (Table 4).

**Table 4:** **Mendelian randomization analysis for the association of genetically raised HDL-C with CAD risk using** ***CETP*** **TaqIB polymorphism as an instrument**

**Meta-analysis of included studies**	**OR**	**95% CI**	**P value** ^**a**^
Per 0.03 mmol/L (1 mg/dL) increase in HDL-C	0.99	0.98–1.00	0.069
Per 0.05 mmol/L (2 mg/dL) increase in HDL-C	0.98	0.96–1.00	0.069
Per 0.10 mmol/L (4 mg/dL) increase in HDL-C	0.97	0.93–1.00	0.069
Per 0.20 mmol/L (8 mg/dL) increase in HDL-C	0.94	0.87–1.01	0.069
Per 0.30 mmol/L (10 mg/dL) increase in HDL-C	0.91	0.81–1.01	0.070
Per 0.50 mmol/L (20 mg/dL) increase in HDL-C	0.87	0.72–1.01	0.071
Per 1.00 mmol/L (40 mg/dL) increase in HDL-C	0.79	0.54–1.03	0.082

^a^: Test for overall effect.

Further, weighted linear regressions of the log odds ratio of genotype-disease have been fitted, against the mean difference in HDL-C level as the explanatory variable, to test the strength of the association between HDL-C and CAD. As a result, there was a statistically significant linear relationship between the standard error of the log odds ratio of the genotype-disease and the mean difference change in HDL-C levels in controls (correlation coefficient = 5.69, P = 0.005). The results suggest that HDL-C indeed lies on the causal pathway between the *CETP* gene and CAD and studies carried out in populations with a large genotype-phenotype difference might be expected to show a large genotype-disease odds ratio.

## Discussion

Because HDL-C serves an important role in CAD pathogenesis, we expected that the *CETP* TaqIB polymorphism, which modulates HDL-C, to likewise confer the risk of disease. To our best knowledge, the current meta-analysis is one of the largest systematic reviews of studies investigating the relationship between the *CETP* polymorphism, HDL-C and potential risk of CAD. The synthesized estimation of the TaqIB polymorphism concerning CAD risk was presented in our meta-analysis containing 50,996 subjects. Subsequently, the possible causal inference of HDL-C on CAD risk was further assessed. The overall OR of the TaqIB polymorphism under a random-effects model was suggestive of a modest protective effect on CAD. Considering that violation of HWE might diminish the total effect of the meta-analysis, we performed a sensitivity analysis by excluding the studies demonstrating a departure from HWE. Trends in the ORs did not change substantially by estimation of the association among only the HWE-confirming studies. We provide robust evidence that the TaqIB polymorphism is associated with CAD and that the HWE-violation only slightly influences the interpretations of the observed results in our meta-analysis. Interestingly, the pre-specified subgroup analyses revealed that the B2 allele carriers had a remarkable higher risk reduction in CAD among Asians compared with Caucasians, in spite of similar frequency of the B2 allele. It remains unclear the real reasons for the divergence across the different ethnic groups, because we did not directly test the TaqIB polymorphism in different ethnic groups. We also did not estimate the association between the TaqIB polymorphism and other phenotypes. Given some GWASs have identified that numerous lipid-associated genetic variations differ with ethnicity [72-74], we expect that future investigations of the TaqIB polymorphism will uncover its genetic ancestral backgrounds and pleiotropic effects. In addition, the magnitude of association was greater in H-B studies than in P-B studies; the OR is likely overestimated, since subjects sampled from a single hospital may not reflect the real environmental exposures in the source population. In theory, participants recruited from the community, or from the general population, are more representative and reliable. Prospective studies and studies regarding MI or ACS as endpoints concerning the association between the TaqIB polymorphism and CAD did not provide robust evidence for a statistically significant association [75]. Of note, the variability of environmental exposures may be another source of between-study heterogeneity, as reflected from our meta-regression analysis. The protective effect of the B2 allele on CAD risk was potentiated in a population with low smoking rates and low circulating HDL-C levels. Although meta-regression reflects an ecological correlation, rather than a causal inference, our results imply that the underlying interactions of the TaqIB genotype with lifestyle and phenotype may influence the development of atherosclerosis. Considering a wide range of confidence intervals of the overall evaluation, further data from large-scale and well-designed studies are required to improve the precise of the study effect.

The strength of the instrument (the TaqIB polymorphism in our study) is determined by the absolute magnitude of its association with the intermediate phenotype (HDL-C in our study). A previous individual patient data-based meta-analysis including 7 large population-based studies and 3 randomized placebo-controlled trials (13,677 Caucasian subjects), have provided robust evidence that the TaqIB polymorphism is significantly associated with HDL among Caucasians [18]. Hence, the TaqIB polymorphism was certified as a qualified predictive instrument when using a Mendelian randomization approach among Caucasians because it correlates with a specific phenotype and consequently impacts the disease in the pathway intermediated by the phenotype. Our results were concordant with two previous meta-analyses including 15,704 and 19,035 participants respectively [18,19]. However, neither of these studies assessed the relationship of HDL-C and CAD after adjustment for TaqIB polymorphism and the triangulation of the TaqIB polymorphism and HDL-C associations with CAD risk remained confounded.

Mendelian randomization, a form of instrumental variable analysis [76], was applied to determine the magnitude of the causal relationship of increased HDL-C with CAD by using the TaqIB polymorphism as the instrument. Interestingly, genetically elevated HDL-C levels did not reduce CAD risk. Our results supplement the evidence challenging conventional views that raising circulating HDL-C levels may translate into protection of CAD risk. Recently, Voight et al. [77] utilized the Asn396Ser polymorphism in the endothelial lipase gene (*LIPG*) and a genetic score containing 14 common single nucleotide polymorphisms (solely concerning HDL-C) as two instruments for Mendelian randomization. As a result, a specific and substantial increase in HDL-C levels due to *LIPG* Asn396Ser and the genetic score was verified to be not related to MI. Employing various instruments may contribute to the precision of instrumental variable estimates and may facilitate examination of potential instrumental variable assumptions. Besides LIPG, CETP was acknowledged to have a profound influence on HDL-C metabolism and consequently CAD risk. The TaqIB polymorphism, a silent base change affecting the 277th nucleotide, is almost fully concordant with another promoter variants −629 C→A [16,78,79]. The data from in vivo studies have documented that the −629 C→A variant is directly functional in regulating the CETP activity [16], suggesting that the TaqIB polymorphism, as a marker of the −629 C→A polymorphism, may be vital in regulating CETP activity and HDL-C metabolism. In order to maximize statistical power, we exploited the TaqIB polymorphism, rather than the −629 C→A polymorphism, as an instrument because extensive data on the TaqIB polymorphism were available. We suggest a strong possibility that raising HDL-C via specific means of inhibiting CETP will not decrease the risk of CAD. Whether CETP efforts pro- or anti-atherogenic effects, by means of the LDL-C receptor pathway, is still unclear [80,81]. As a matter of fact, the results from animal studies and cytological studies remain paradoxical. Transgenic mice expressing CETP demonstrate accelerated atherosclerosis compared with non-expressing controls [82-84]. However, another in vivo study reported that CETP expression reduced atherosclerosis in lecithin cholesterol acyltransferase (LCAT) transgenic mice [85] and a further study revealed that CETP offset the deleterious effect of LCAT [86].

Furthermore, we restate the underlying restriction of raising circulating HDL-C levels as an effective treatment to reduce residual cardiovascular risk in patients treated by conventional statin therapy. Intense research efforts on therapeutic agents have been conducted to reduce the residual cardiovascular risk by raising circulating HDL-C levels. Statins, fibrates and niacin are regarded as effective pharmacological agents for raising HDL-C. However, the Action to Control Cardiovascular Risk in Diabetes (ACCORD) study documented there was no added clinical benefit in using combination therapy with fenofibrate plus simvastatin compared with simvastatin monotherapy, despite the fact there were significantly increased levels of HDL-C in the combination therapy group [5]. In the Atherothrombosis Intervention in Metabolic Syndrome with Low HDL/High Triglycerides: Impact on Global Health Outcomes (AIM-HIGH) trial, the addition of niacin to statin therapy did not reduce the rate of cardiovascular events, despite remarkable improvements in HDL-C [87]. The Investigation of Lipid Level Management to Understand its Impact in Atherosclerotic Events (ILLUMINATE) trial exhibited an excess of deaths and cardiovascular disease in the group receiving the CETP inhibitor, torcetrapib and atorvastatin compared with atorvastatin alone [8]. Subsequently, another large randomized trial performed in ACS patients showed that another CETP inhibitor, dalcetrapib, increased HDL-C levels, but did not reduce the risk of recurrent cardiovascular events [9]. Recently, a potential explanation was put forward that functional HDL-C properties may play a key role on the development of CAD rather than an indirect one [88]. Cholesterol efflux and reverse cholesterol transport is an important function of HDL-C. In vitro experiments and transgenic animal model studies have revealed that circulating HDL-C concentrations do not necessarily reflect the efficacy and anti-atherogenicity of reverse cholesterol transport [89]. It is likely that the increments of HDL-C generated by the CETP inhibition are either non-functional or pro-inflammatory rather than anti-inflammatory, which impedes their physiologic role in reverse cholesterol transport.

Another possibility is that the composition of HDL-C is altered in CAD patients and they are no longer productive at high levels or after therapeutic intervention [9]. Some studies have indicated that HDL-C and its major structural protein, apolipoprotein A1 are dysfunctional and are extensively oxidized by myeloperoxidase in human atheroma. In vitro oxidation of either apolipoprotein A1 or HDL particles by myeloperoxidase impairs their cholesterol acceptor function [90]. The precise evaluation of functional HDL-C properties and change in HDL-C sub-fractions are recommended for future studies.

Common variants in lipid-associated loci that are also associated with CAD may implicate genes at these loci as possible therapeutic targets. Recently, a large meta-analysis based on GWAS aimed to investigate the genetic markers for blood lipids [91]. A genome-wide association screen for serum lipids (including approximate 2.6 million SNPs) was conducted in 100,000 individuals of European ancestry. Ninety-five loci (of which 59 are novel) were identified to show genome-wide significant association with serum lipid traits. Further, the lead SNPs from the study were tested in 24,607 CAD patients and 66,197 individuals without CAD. They documented that common variants in lipid-associated loci are consistently related to CAD, implying that these loci may be the potential therapeutic targets. They also suggested that some causal genes in lipid-associated loci may have pleiotropic effects on non-lipid parameters that play an important role on the CAD risk reduction. They provided a foundation from which to develop a broader biological understanding of lipoprotein metabolism and to identify potential therapeutic targets for preventing CAD.

There are still limitations using Mendelian randomization analysis. Potential pleiotropic effects of genes may influence the results when a naturally occurring single nuclear polymorphism is used as an instrument to evaluate disease causality. Although single genetic variation is relative to extensive diseases, many of these associations are false positives because the gene has been more generally characterized than most in population investigations of genetic association studies. Canalization or developmental compensation also provides difficulties in interpreting the gene-disease association. These processes, which bring developmental buffering, offset the influence of the genetic variation or environmental forces [92], remain difficult to evaluate. Further experimental and subject-matter studies may provide a more precise estimation. In addition, CAD has numerous clinical features, ranging from stable coronary syndrome to ACS, which is determined by the stability of the atherosclerosis plaque. We could not completely exclude noise in our data when using CAD as the endpoint. Considering more power would be obtained from a more refined phenotype than a more global one, a more precise method that identifies the vulnerable plaque is needed and may be beneficial to estimate the effect of HDL on coronary atherosclerosis.

## Conclusion

In conclusion, our results provided robust evidence that the TaqIB polymorphism, which specifically raised HDL-C concentrations, was uniformly associated with a reduction in CAD risk. However, a lack of causal inference in increased HDL-C levels with the pathogenesis of CAD was detected based on the Mendelian randomization approach among Caucasians. We highlight not only the requirements for further validation of the functional role of HDL-C in reverse cholesterol transport and in the etiology of atherosclerosis, but also as a high possibility that lifestyle interventions or pharmacotherapy, which elevates circulating HDL-C levels, cannot be assumed to benefit CAD patients in reality.

## Additional files

Additional file 1: Table S1. The baseline characteristics of all eligible studies in the meta-analysis.

Additional file 2: **Supplementary material.** Study descriptions.

## Abbreviations

HDL-C: High density lipoprotein cholesterol
CAD: Coronary artery disease
CETP: Cholesteryl ester transfer protein
SD: Standard deviation
MI: Myocardial infarction
LDL-C: Low density lipoprotein cholesterol
VLDL-C: Very low density lipoprotein cholesterol
CNKI: China nation knowledge infrastructure platform
CBM: China biological medicine
ACS: Acute coronary syndrome
MONICA: Monitoring of trends and determinants in cardiovascular disease
BMI: Body mass index
HWE: Hardy-Weinberg equilibrium
OR: Odd ratio
SMD: Standard mean difference
CI: Confidence interval
H-B: Hospital-based
P-B: Population-based
NHS: Nurses’ Health Study
HPFS: Health Professionals follow-up study
LCAT: Lecithin cholesterol acyltransferase
ACCORD: Action to control cardiovascular risk in diabetes
AIM-HIGH: Atherothrombosis intervention in metabolic syndrome with low HDL/High triglycerides
ILLUMINATE: Investigation of lipid level management to understand its impact in atherosclerotic events
GWAS: Genome-wide association studies
