# Supplementary material for: Association of cholesteryl ester transfer protein (CETP) gene polymorphism, high density lipoprotein cholesterol and risk of coronary artery disease: a meta-analysis using a Mendelian randomization approach
Source: BMC Med Genet. 2014 Oct 23;15:118. doi: 10.1186/s12881-014-0118-1 (PMC4258818; doi:10.1186/s12881-014-0118-1)
Supplement: Supplementary file 2 — Supplementary material. Study descriptions. [file 12881_2014_118_MOESM2_ESM.docx]

**Study Descriptions**

**1. The study by Arca M et al** A case-control study performed in a cohort of Italian subjects, including 415 subjects with angiographically documented coronary artery disease (CAD), 215 subjects without CAD(non-CAD) confirmed by coronarography and 188 healthy individuals (randomly selected from a community based screening for coronary risk factors). One hundred and sixty-four CAD patients had a history of myocardial infarction (MI). Three hundred and sixteen CAD subjects provided clinical records allowing diagnosis of previous ischaemic complications. CAD was defined as one or more >50% stenoses in at least one major coronary artery. Non-CAD was defined as <10% stenoses. Angiographically assessed patients and population controls were characterized as previously reported [[1](#_ENREF_1)].

**2. The study by Bhanushali et al** The study was performed on a total of 240 unrelated Indians which consisted of 90 patients proven CAD. The other 150 were clinically free from CAD and acted as controls. CAD is confirmed by coronary angiography: >50% stenosis in one or more arteries and stable or unstable angina. Controls are examined clinically and investigated by electrocardiography to exclude CAD. The detailed information on demographics, medical history, coronary risk factors and current medication was recorded through personal interviews and through their medical records.

**3. The study by** **Blankenberg et al (The AtheroGene Case-Control Study)** The details of study design of the AtheroGene Study has been previously described[[2](#_ENREF_2)]. In general, cases and controls were of German nationality and were inhabitants of the Rhein-Main area. Between November 1996 and July 1998, 1214 CAD patients underwent coronary angiography at the Department of Medicine II of the Johannes Gutenberg- University Mainz or the Bundeswehrzentralkrankenhaus Koblenz. CAD is defined as a stenosis greater than 30% in at least 1 major coronary artery. Healthy control subjects (n=574) were recruited either from general practitioners’ offices in the course of routine check-up visits or by newspaper announcement. This announcement invited healthy individuals aged ≥40years to participate as control subjects. The individuals without any clinical or anamnestic evidence of a history of atherosclerosis and without evidence of any pathological [electrocardiogram](javascript:void(0);) pattern were selected.

**4. The study by Corella et al (European Prospective Investigation into Cancer and Nutrition [EPIC] cohort study)** A nested case-control study among participants of the Spanish EPIC cohort study was performed. EPIC is a large prospective study conducted in 10 European countries. The Spanish branch of EPIC including 41,440 healthy individuals (15,632 men and 25,806 women) aged 30–69 years. They were recruited between October 1992 and July 1996 in five Spanish regions (Asturias, Navarra, Gipuzkoa, Murcia and Granada) and were followed up over a 10-year period [[3](#_ENREF_3)]. Cases were defined as fatal or non-fatal MI or angina requiring a revascularization procedure. Medical records of all potential cases were reviewed by trained personnel. Each participant was administered questionnaires to collect information on demographic characteristics, lifestyle and medical history in face-to-face interviews. Until the end of 2004, a total of 719 cases including 459 fatal and non-fatal MI cases, 141 angina cases and 119 probable or possible MI cases were identified. Two controls were matched to each case by center, sex, age (within 5 years), and time of enrolment (within 3 months). In the current study, 557 CAD patients (non-fatal acute MI [n = 422], extra-hospitalized fatal acute MI [n = 8] and angina pectoris requiring revascularization procedure [n = 127]) and 1180 healthy controls were analyzed. Controls were considered as those subjects who remained free of CAD after the 10-year follow-up period.

**5. The study by Dedoussis et al ( the CARDIO2000-GENE Study)** Detailed information of the CARDIO2000-GENE study have been previously published [[4](#_ENREF_4)]. Between June 2002 and December 2002, 237 hospitalized patients (185 males) with a first event of an acute coronary syndrome (ACS) were selected in regions across Greece. Participating patients did not have a history of CAD (e.g. stable angina). Medical information was retrieved from the hospitals’ or insurance records. Socio-demographic background factors and lifestyle data was obtained using a confidential questionnaire. ACS included acute MI and unstable angina. Two hundred and thirty-seven subjects (185 males) without any clinical symptoms or history of cardiovascular disease, matched to the patients by age, sex, and region were randomly selected. The controls were surgical patients of the same hospital during the same period as the ACS patients.

**6. The study by Durlach et al** The cross-sectional study comprised 406 unrelated French patients with noninsulin-dependent diabetes (NIDDM) (231 men and 175 women), aged 59.5±10.8 yr (27–83 yr), from the Diabetes Center in Reims. This study aimed to investigate micro- and macro-vascular complications of NIDDM. The criteria for noninsulin-dependent diabetes were those defined by the National Diabetes Data Group. Mean duration of diabetes was 11.7±7.7yr.The patients were treated by diet and/or antidiabetic drugs. Eighty-seven patients were under lipid lowering. Nineteen percent of the patients were smokers. Most women were postmenauposal and none received hormonal replacement therapy. CAD included previous MI and/or angina. Arteriopathy was diagnosed on the basis of intermittent claudication or absence of peripheral pulse confirmed by ultrasonography. Retinopathy was diagnosed by fundoscopy and angiography.

**7. The study by Eiriksdottir et al (the Reykjavik study)** This is a prospective population**-**based study surveying cardiovascular diseases and their risk factors. This study has been on-going since 1967, as described elsewhere [[5](#_ENREF_5)].

The study has been conducted in six different stages at about 5 year intervals. Two thousand men residing in the Reykjavik area were invited to participate in all stages and randomly assigned to different study groups. The current study comprised non-fatal MI cases (388 male) and a control group of 794 healthy men (participants in the sixth stage). All known survivors of MI in the Reykjavik Study were regarded as cases. The mean age of this group was 71 years and the average time after the MI event was 11 years.

**8. The study by Falchi et al** The case-control study including 100 unrelated individuals with angiographically documented CAD (85 males and 15 females, mean age 46 ± 0.03 years) and 100 unrelated healthy blood donors (60 males and 40 females, age 37±0.04 years) in isolate Central Corsican subpopulations (Corte, Niolo and Bozio) [[6](#_ENREF_6)]. The samples were selected at Bastia Hospital.

**9. The study by Freeman et al (the West of Scotland Coronary Prevention Study [WOSCOPS])** WOSCOPS was a primary prevention study that demonstrated a significant reduction of cardiovascular morbidity and mortality by pravastatin treatment. Baseline characteristics of the WOSCOPS subjects have been described previously [[7](#_ENREF_7)]. The WOSCOPS cohort comprised

6,595 males aged 45–65 (mean 55.2) years and 580 cardiovascular events (fatal and non-fatal) occurred during the 5-year course of the study. Recruits had no history of MI and had normal renal and hepatic function. Cases are defined as a definite or suspect fatal or non-fatal MI, sudden coronary death or required coronary artery bypass graft (CABG) or angioplasty. The current study is a nested case-control study. Each patient was matched with two controls (also drawn from the original cohort). A proportion of samples (6.7%, n=116) were missing from the Biobank and not available to prepare DNA.

**10. The study by Fumeron et al (the Etude Cas-Témoin de l’Infarctus du Myocarde [ECTIM] study)** The ECTIM study is a large case-control study of MI that was set up to investigate the large differences in CAD incidence and mortality between Strasbourg (Eastern France), Toulouse (Southwestern France), Lille (Northern France) and Belfast (Northern Ireland). All participants were white Europeans. They had to be residents of the region, and their parents and grandparents had to have been born in Europe (for the French centers) or the historical entity of Ulster (Northern Ireland). Male patients aged 25-64 who survived an MI (MONICA category I) were eligible. The patients were drawn from the MONICA registers, and further epidemiological data and blood samples were obtained at least 3 months and at most 9 months after the event. Male age-matched controls were randomly recruited from the same areas and were obtained from the electoral rolls in France and from the lists of general practitioners held by the Central Services Agency in Northern Ireland. The subjects were examined in clinics or, if necessary, at home by specially trained staff. A set of questionnaires was completed which included details of personal history, presence of disease, drug intake, cigarette smoking and alcohol consumption. Information on CETP/TaqIB genotype, alcohol consumption, and plasma HDL cholesterol was available from 608 patients and 724 controls.

**11. The study by Horne et al (the Intermountain Heart Collaborative Study)** Between August 1994 and June 2004, a cohort of 10,020 patients undergoing angiography were enrolled in the cardiac catheterization registry of the Intermountain Heart Collaborative Study. CAD was regarded as the primary study end point. MI and lipid levels were evaluated as secondary end points. The MI events were determined from Intermountain Healthcare’s electronic record repository and were ruled-in by electrocardiography and/or biomarker measurements. The current study population included 4,811 non-diabetic non-smoking patients undergoing coronary angiography at hospitals within the Utah-based Intermountain Healthcare system. Patients were categorized as being free of CAD (i.e., free of CAD or with minimal, <10% stenosis), having moderate CAD (i.e., most severe lesion 10% to 69% stenosis), or having significant CAD (i.e., ≥1 lesion of ≥70% stenosis). The group of moderate CAD was excluded. CAD presence was defined as those with significant CAD, and those patients free of CAD. Demographic and health history data were obtained from physicians and hospital records and were stored in a research database.

**12. The study by Hsieh et al** A case-control study involving 365 Taiwanese with type 2 DM recruited from the diabetic clinic in the Metabolism Division at Kaohsiung Medical University Hospital, Kaohsiung, Taiwan. The diagnosis of diabetes was based on American Diabetes Association criteria. Among these DM patients, 101 patients with CAD were diagnosed according to a clinical history of angina pectoris/ MI and were confirmed by coronary angiography. Two hundred and sixty-four DM patients without CAD had a normal electrocardiogram and no history or clinical signs of CAD, based on a maximal negative exercise test. Individual interviews were held with patients about their disease and smoking history. Patients received a complete physical examination as well as an assessment for the presence and extent of macro- or micro-vascular complications.

**13. The study by Izar et al (The genetics, outcomes, and lipids in type 2 diabetes [GOLD] study)** A prospective, multi-center study was conducted among 990 type 2 DM patients (aged 35-80 years) recruited from 27 institutions in Brazil. Diabetic cases (n = 386) were collected using physician confirmed diagnosis of MI, while the control group (n=604) consisted of diabetic patients without MI and also any clinical manifestation of arterial disease. Myocardial infarction status was assessed using the WHO criteria.

Demographic data, physical examination, and a standard 12-lead electrocardiogram were obtained locally. The electrocardiograms were examined in a blinded manner by two independent investigators unaware of the patients’ condition. The presence of left ventricle hypertrophy was tested by Perugia’s criteria.

**14.** **The study by Jensen et al (the Health Professionals Follow-up Study [HPFS] and the Nurses’ Health Study [NHS])** The Parallel nested case-control studies in the NHS and the HPFS where 246 women and 259 men who developed incident CAD were matched to controls (1:2) on age and smoking. The NHS cohort was initiated in 1976 at the Channing Laboratory of the Brigham and Women’s Hospital. The study population consists of 121, 700 married female registered nurses aged 30–55 years residing in one of 11 larger US states. Women were biennially contacted to update information on exposures and newly diagnosed illnesses on follow-up questionnaires. Since 1980, participants have updated information on diet, alcohol, and vitamin supplements through a food frequency questionnaire approximately every 4 years. The HPFS was initiated in 1,986 at the Harvard School of Public Health, when 51,529 male health professionals (29,683 dentists, 3,745 optometrists, 2,218 osteopathic physicians, 4,185 pharmacists, 1,600 podiatrists, and 10,098 veterinarians) aged 40–75 years from throughout the US completed the initial questionnaire. The update information is obtained biennially in a manner similar to the NHS.

**15. The study by Kaestner et al** A prospective study included 204 Greek patients with CAD (178 men and 26 women) undergoing percutaneous trans luminal coronary angioplasty (PTCA) between 2000 and 2002. Thirty-five healthy, age- and gender-matched controls in the same population were collected in 2002. These CAD patients had been receiving statins at different doses for different time periods. Information about smoking habits, alcohol intake, hypertension, total plasma cholesterol levels, and any family history of CAD was obtained for all patients. Patients were followed up clinically and by treadmill tests and/or thallium scintigraphy at follow-up visits 3 and 6 months after the PTCA procedure.

**16. The study by Kawasaki et al** A cross-sectional study included 443 Japanese patients with type 2 DM (248 men and 195 women) selected randomly among patients who were admitted to the diabetes center at Osaka City University Hospital from April 1997 to February 2000. This study aimed to investigate the association between the *CETP* gene polymorphism and the incidence of macro-vascular disease. The diagnosis of DM was based on a previous history of diabetes or on American Diabetes Association criteria. The diagnosis of CAD was based on symptoms and clear ischemic changes in an electrocardiogram, either at rest or during an exercise test, or on findings from coronary angiography.

**17. The study by Keavney et al (The International Study of Infarct Survival [ISIS])** The design of the ISIS genetic study has been described in detail [[8](#_ENREF_8)]. The ISIS-3 involved a randomized trial that compared the effects of different fibrinolytic regimens and of different antithrombotic regimens started soon after admission to hospital in nearly 46, 000 patients presenting within 24h of the onset of suspected acute MI. Baseline information was collected on each patient during the central telephone randomization procedure, including details of the presenting electrocardiogram and of any history of previous MI. Additional information was obtained at discharge from hospital (or earlier death) on whether major clinical events had occurred during the patient’s hospital stay. A few months after hospital discharge, information was sought from surviving cases concerning various aspects of their lifestyle. The current study involved 4,685 cases and 3,460 unrelated controls. Cases were males aged 30–54 years and females aged 30–64 years, with MI confirmed by cardiac enzyme and/or electrocardiographic criteria. Controls were aged 30–64 years with no history of MI, angina, or other definite heart disease. The present study involved only those controls who were not first-degree relatives of cases (i.e., spouses only).

**18. The study by Kolovou et al** A case-control study included 471 subjects from various parts of Greece. Subjects were consecutively recruited among those admitted to hospital for coronary angiography. The Onassis Cardiac Surgery Center is a major referral hospital for cardiac disorders. The CAD patients were angiographically documented and were classified as left main coronary artery disease (LMCAD) group (n = 133), more peripheral coronary artery disease (MPCAD) group (n = 241) and control group (n = 97). LMCAD was defined as a lesion compromising the lumen by >30% proximal to the bifurcation, including ostial stenosis. MPCAD was defined as lesions compromising the lumen by >50% further from LM. Control group was recruited from Onassis Cardiac surgery Center personnel and teachers from TEI schools (without major risk factors) who were self reported as healthy.

**19. The study by Li et al** A case-control study involved 290 Chinese subjects undergoing coronary angiogram between January 2005 and December 2006. Two hundred and thirty-six ACS patients were diagnosed according to cardiac enzyme and electrocardiographic criteria and confirmed by coronary angiography ((i.e., ≥1 lesion of ≥70% stenosis, [thrombus](javascript:void(0);) or unstable plaque). The control group included 54 unrelated individuals with normal coronary arteries as also determined by angiography.

**20. The study by Liu et al (the Physicians’ Health Study [PHS])** The PHS was a prospective, randomized, double-blind, placebo-controlled trial designed to estimate the effects of aspirin and beta-carotene on the primary prevention of cardiovascular disease and cancer [[9](#_ENREF_9)]. 14,916 male physicians initially free of cardiovascular disease were recruited. During an average of 9 years of follow-up, 384 men had an MI, confirmed by medical records. The current study was a nest case-control study among these MI patients and among an equal number of age and smoking-matched participants. Controls were randomly selected from study participants who remained free of cardiovascular disease during follow-up.

**21. The study by McCaskie et al** This study included three Western Australian populations. Among these, 2 were cross-sectional community-based populations ( the Carotid Ultrasound Disease Assessment Study [CUDAS] and the Busselton Population Health Survey) and the other study ( the Carotid Ultrasound in Patients with Ischaemic Heart Disease [CUPID] ) included 556 CAD patients. These three populations were predominantly European-Australian and were collected from the same region of Western Australia. The CUDAS group consisted of 1,109 subjects randomly selected from an electoral survey in the Perth metropolitan area. This study has an equal male to female ratio and equal numbers in each age decile. The Busselton Population Health Survey cohort consisted of 1,574 subjects and was studied as part of a cross-sectional community study in 1994/95. The patients of the CUPID study received angiographic assessment in Sir Charles Gairdner Hospital, Perth. CAD was diagnosed as least one coronary vessel with >50% stenosis.

**22. The study by Meiner et al** A case-control study included subjects residing in three contiguous counties of western Washington State. Cases were diagnosed with a first fatal or nonfatal MI by screening the hospital discharge diagnoses provided by all hospitals within the study region between July 1998 and December 2000 (women) and July 1998 and March 2000 (men). MI was defined by evidence of symptoms, elevated enzymes, and electrocardiographic changes. As a result, 578 (257 women and 321 men) were contacted to participated in an in-person interview, delivering an overall case response rate of 54.9%. A random-digit telephone dialling was used to identify a control group residing in the same area during the time period of the study. 666 controls frequency-matched on age were identified. The estimated response rate, incorporating both the household screening and interview participation rates, was 54.7%. All subjects were interviewed using a structured questionnaire for the time period preceding the MI or an equivalent date for controls.

**23. The study by Mohrschladt et al** A prospective study comprised 400 familial hypercholesterolemia patients, who received and responded to a questionnaire (response rate 96%) on cardiovascular events after intake at the Lipid Clinic from the Leiden University Medical Center. Three hundred statin-treated patients (143 male and 157 female patients, mean age 48 years) were included in the present study. Details of the methods were described earlier [[10](#_ENREF_10)]. All patients received a routine physical examination. Cardiovascular disease (CVD) was designated as angina pectoris, MI, intermittent claudication (CI), PTCA, CABG and arterial peripheral vascular surgery. CVD was present at baseline in 39% of the patients. All information on events was confirmed by tracking hospital records.

**24. The study by Muendlein et al** A case-control study was conducted among 560 consecutive Caucasian patients referred to coronary angiography for evaluation of CAD, as described previously [[11](#_ENREF_11)]. A standardized interview was used to obtain information on cardiovascular risk factors. Coronary angiography was performed with the Judkins technique. CAD was diagnosed in the presence of significant coronary stenoses with lumen narrowing of at least 50%.

**25. The study by Padmaja et al** A case-control study was conducted in Pharmacogenomics lab, Department of Pharmacology in collaboration with Department of Cardiology between June 2004 and June 2007. Five hundred and four unrelated electrocardiograph confirmed cases of CAD, recruited from cardiology clinics, JIPMER and 338 individuals, matched by age and sex, belonging to the Tamilian population of south India were studied. The control patients were recruited in medical and surgical clinics of the same hospital during the same study period. All of the control patients had no history of CVD and were recruited carefully after examination for clinical symptoms of CAD and electrocardiograph confirmation. Both the cases and controls were of Tamilian origin as they were all residents of Tamilnadu or Pondicherry for at least three generations. The cases, defined as MI or unstable angina were confirmed by clinical diagnosis based on coronary angiogram/ECG findings/Tread Mill test/Echo. Data on lifestyle factors was collected using an interviewer-administered questionnaire. The questionnaire included details of personal history, presence of disease, drug intake, cigarette smoking, and alcohol consumption.

.

**26. The study by Park et al** This case-control study involved 119 CAD patients and 106 controls in Koreans. All participants had no history of lipid-lowering therapy and were enrolled between February 2001 and July 2001 at Seoul National University Hospital (SNUH). CAD was defined as a luminal narrowing of >50% in at least one coronary artery, as judged by coronary angiography. Controls were those who had normal or insignificant coronary angiographic findings, or patients with normal electrocardiograms who did not have clinical symptoms/signs of CAD. The presence of CAD risk factors was recorded.

**27. The study by Poduri et al** This study recruited unrelated 265 CAD patients of North Indian ethnicity in Cardiology Clinics of Nehru Hospital, PGIMER, Chandigarh. CAD was confirmed by coronary angiogram (>50% stenosis). Lipid-lowering drugs were stopped for 2 weeks for patients who were on these drugs to allow a wash out period. One hundred and fifty individuals with normal [electrocardiogram](javascript:void(0);) and negative stress test and who had no family history of ischemic heart disease or any other disease were age-, sex-, and ethnicity matched to the cases. Patients and controls were ascertained to have at least both parents and grandparents born in North India (Punjab, Haryana, and Chandigarh). Cases including 230 MI and 35 non-MI patients were documented by review of medical records including history, ECG, enzyme changes, and/or the typical sequelae of MI on ventricular angiography.

**28. The study by Porchay-Balderelli et al (the Noninsulin-Dependent Diabetes, Hypertension, Microalbuminuria, Proteinuria, Cardiovascular Events, and Ramipril [DIABHYCAR] study)** This was a multi-centric, random, double-blind, parallel group trial to compare the cardiovascular and renal outcomes of patients taking ramipril and those taking placebo[[12](#_ENREF_12)]. All participants with type 2 DM were aged ≥50 years, with serum creatinine ≤150μmmol/L and urinary albumin excretion≥20mg/L. In the current study, only 3,124 French patients were included. The average duration of follow-up was 4 years. All participants were examined every 6 months for at least 3 years. CAD was defined as a combination of MI and sudden death. Incident MI was defined as the first occurrence of a fatal or nonfatal MI after the baseline examination. Sudden death was defined as death occurring instantaneously or within 1 h after the onset of new cardiac symptoms (arrhythmia or other cardiovascular causes) or non-witnessed death, where the body of the deceased was found, and no cause could be discovered.

**29. The study by Qin et al** A case-control study was performed in a Chinese population between January 2002 and July 2003. Two hundred and forty-nine individuals ( mean age 58.81±8.85 years) were angiographically documented CAD patients while 167 subjects without CAD (excluded by coronary angiography, mean age 55.11± 8.96 years ) were regarded as controls. CAD was defined as a luminal narrowing of >50% in at least one major coronary artery. Hypertension, DM and hemopathy were excluded among controls by a series of clinical examination.

**30. The study by Rahimi et al** All participants were from a western population of Iran with Kurdish ethnic background and underwent elective angiography in the Cardiology Division of the Imam Ali Hospital of the Kermanshah University of Medical Sciences. Patients were subdivided into three groups in this study: Two hundred and seven CAD patients (113men and 94 women, mean age 56.9±8.6 years), 101 unrelated type 2 DM patients (51men and 50 women, mean age 56.5±9.8 years) and the control group involving 92 unrelated subjects (47 men and 45 women, mean age 54.3±8.5 years). The controls consisted of non-diabetic individuals who had normal coronary arteries. CAD was defined as ≥50% diameter obstruction of a major coronary vessel. All films were reviewed by two cardiologists with no previous knowledge of the condition of the patients.

**31. The study by Rejeb et al** A case-control study including 316 patients undergoing coronary angiography in the Cardiology Department at Sahloul University Hospital, Sousse, Tunisia between 2003 and 2007. Two hundred and twelve CAD patients had significant coronary artery stenosis, which was defined as a luminal narrowing ≥50% in at least one major coronary artery, as judged by coronary angiography. One hundred and four controls had normal or insignificant coronary angiographic findings (< 50%). Data on lifestyle factors were collected using an interviewer—administered questionnaire. The questionnaire included details of personal history, presence of disease, drug intake if any, smoking and alcohol consumption. Patients taking lipid-lowering drugs were excluded.

**32. The study by Schierer et al (Sikh Diabetes Study [SDS])** The study population is a part of the ongoing the SDS [[13](#_ENREF_13)]. The study was performed in 2,431 participants drawn from a unique Sikh population of Northern India (Punjab). Individuals of South, East, and Central Indian origin were excluded. In the current study, 1,307 type2 DM cases and 1,124 normoglycemic controls were included. Questionnaires provided information on demographic characteristic and life style of all the participants. In general, Punjabi Sikhs do not smoke for religious and cultural reasons. Approximately 50% of participants were life-long vegetarians, and the vast majority of Punjabi females do not drink alcohol. Information regarding CAD, date of CABG or angioplasty, and medication usage was obtained from patient records. CAD was diagnosed based on nitrate medication records, electrocardiographic, angiographic or echocardiographic evidence. About 15% of participants had CAD.

**33. The study by Tenkanen et al** The study population was of Finnish origin. The population sample involved 187 hyperlipidemic patients who were from the Helsinki Heart Study (a randomized placebo-controlled coronary primary prevention trial with gemfibrozil in middle-aged hyperlipidemic men) and 111 unrelated healthy individuals (50 men and 61 women) were included. Among 187 hyperlipidemic men, 72 had suffered a MI during the 5-year trial. The lipid data were available in the second screening visit 2-3 months before treatment was initiated.

**34. The study by Van Acker et al (the Regression Growth Evaluation Statin Study, REGRESS)** The present study comprised two groups of subjects, a cohort of male patients with angiographically documented CAD in the REGRESS and a group of age-matched, nonsymptomatic population controls. The REGRESS, described in detail elsewhere [[14](#_ENREF_14)], was a randomized, placebo-controlled, multi-centre study to assess the effect of 2 years of pravastatin treatment on the progression and regression of coronary atherosclerosis under the auspices of the Interuniversity Cardiology Institute of The Netherlands. 885 Caucasian men with angiographically confirmed CAD (>50% stenosis of at least 1 major vessel) were included in the study. All patients were < 70 years of age, had total cholesterol levels between 4 and 8 mmol/L, and had triglyceride levels < 4 mmol/L. The patients were randomized to receive pravastatin 40 mg once daily or matching placebo. Controls were non- CAD subjects, normolipidemic healthy males with Dutch nationality and were randomly selected from the population-based Monitoring Project on Cardiovascular Disease Risk Factors. The control subjects had no history of CVD, DM or cancer and did not take medication of hypertension or hypercholesterolemia or anticoagulants. Total cholesterol levels were required to be between 4 and 8 mmol/L, whereas the body mass index was required to be <33 kg/m^2^.

**35. The study by Wang et al (2008)** The study included 186 patients receiving coronary angiography. All participants were drawn from a Han population of Southern China (Ningbo). One hundred and eleven CAD patients were diagnosed as one or more >50% stenoses in at least one major coronary artery. Seventy-five controls had any lumen stenoses <10%. Stroke, peripheral vascular diseases or taking lowing-lipid drugs within 2 weeks prior to admission were excluded.

**36. The study by Wang et al (2004)** This case-control study included 128 CAD patients, aged 62.9±9.6 years and 247 unrelated controls without CVD, aged 59.5±8.4 years. All subjects were of Chinese Han nationality and were recruited in Hubei between October 2002 and May 2003. CAD was defined as a luminal narrowing ≥50% in at least one major coronary artery.

**37. The study by Whiting et al (the Intermountain Heart Collaborative Study [IHCS])** The study patients was drawn from a population of 4,704 individuals who underwent coronary angiography in the IHCS Cardiac Catheterization Laboratory Registry [[15](#_ENREF_15)]. All participants were drawn from a population that is primarily White of Northern European extraction, shown previously to be genetically similar to the general North American Caucasian population. CAD was defined as a diameter stenosis of ≥70% in ≥1 coronary artery or its major branch and was further divided into 3 subgroups (1-, 2-, or 3-vessel CAD). Controls had normal coronary arteries (no lesions visible) confirmed by coronary angiogram. Mild/moderate disease (10%-60% stenosis of ≥1 coronary artery) was excluded. Angiographic assessment was made by experienced angiographers with no knowledge of genotyping. The degree of maximal diameter stenosis for each of the 3 principal coronary arteries and their major branches was estimated visually to the nearest 10% and entered into the IHCS Registry database.

**38. The study by Wu et al** The case-control study included 200 CAD patients (138 males and 62 females, 28–83 years old) and 285 control subjects (155 males and 130 females, 20–82 years old). The CAD patients were survivors of acute MI and were diagnosed by cardiac catheterization (greater than 70% stenosis in the left descending left circumflex or right coronary artery and greater than 50% stenosis in the left main coronary artery). The controls without risk factors for CAD had a normal resting electrocardiogram and Master’s exercise test in physical examination. All subjects were Taiwanese.

**39. The study by Yan et al** All participants in the current study were drawn from a Northern Han Chinese Population. One hundred and six CAD patients ( 81 males and 25 females) were diagnosed as significant coronary stenoses with a lumen narrowing ≥50% in at least one major coronary artery. The control group comprised 64 unrelated individuals with normal coronary arteries confirmed by coronary angiogram. DM, nephropathy, hepatopathy, thyroid and adrenal diseases were excluded.

**40. The study by Yang et al** This study was performed in a Chinese population. This study included 3 groups of subjects: the control group involving 92 volunteers from healthy individuals or medical staff, 71DM patients without CAD (age 61.7±6.1years) and 83 type2 DM patients with CAD (age 64.3±6.4years). The duration of DM was longer than that of CAD. The clinical data was recorded in detail.

**41. The study by Yilmaz et al** The case-control study comprised 173 CAD patients (31.2% women and 68.8% men) and 111 healthy volunteers (41.9% women and 58.1% men). The severity of CAD was estimated by coronary angiography. CAD was defined as ≥50% stenosis of at least one major coronary vessel. Vascular events were defined as MI, PTCA and CABG. Healthy persons without any history of vascular events had not any symptoms of CAD. However, coronary angiography was not performed on these individuals, and therefore the presence of atherosclerotic coronary arteries could not be excluded.

**42.** **The study by Zhang et al (2005)** This study included 88 Chinese patients undergoing coronary angiography. Thirty-eight patients had significant coronary stenoses with lumen narrowing of at least 50% and were diagnosed as CAD (age 65±10 years). The control group comprised 67 healthy individuals and 27 patients with normal coronary arteries (also confirmed by coronary angiogram). The control group had no history of CAD and DM (age 54±18 years).

**43. The study by Zhang et al (2011)** All participants in the current study were of Chinese Han nationality and were inhabitants of Hainan province. Three hundred and thirty-four CAD patients and 301 unrelated healthy controls were recruited. CAD was defined as a lumen narrowing ≥50% in at least one major coronary artery. The control group had no history of CVD, DM and hemopathy. There was non-significant difference between cases and controls in age, sex and weight.

**44. The study by Zhao et al** The study recruited a Chinese population (224 men and 217 women, age 61.3±9.2 years) between July 2000 and September 2002. The CAD group (n= 238) comprised 54 acute MI, 106 unstable angina pectoris, 35 stable angina pectoris and 43 old MI patients. Among these CAD patients, 57 were documented by coronary angiography. Two hundred and three controls without CAD were recruited either from hospitalized patients or routine check-up visits.

**45. The study by Zheng et al** The study population was recruited from West China Hospital, Sichuan University for the study from April 2001 to March 2002. The CAD group including 203 patients (137 men and 66 women, aged 36 - 84 years) was selected consecutively based on coronary angiography. CAD was defined as a stenosis of more than 50 % in any major coronary artery branch (left anterior descending artery, left circumflex artery, right coronary artery). The CAD group included 117 patients with angina pectoris, 46 with prior MI and 40 with acute MI. The control group consisted of 100 unrelated age- and gender- matched subjects (66 males and 34 females, age 41 - 69 years). There was non-significant stenosis found by angiography among controls.

**46. The study by Zhou et al** All participants involved in the study were of Chinese Li nationality. They were ascertained to have both parents and grandparents of Li origin in Hainan Province. Forty-seven CAD patients (22 males and 25 females, age 60±12 years) and 330 healthy individuals ( 178 males and 152 females, age 62±9 years) were included. CAD was defined as a lumen narrowing ≥50% in at least one major coronary artery. The controls had no history of DM, CVD and hemopathy.

**Reference**

**1. Baroni MG, D'Andrea MP, Montali A, Pannitteri G, Barilla F, Campagna F, Mazzei E, Lovari S, Seccareccia F, Campa PP *et al*: A common mutation of the insulin receptor substrate-1 gene is a risk factor for coronary artery disease. *Arterioscler Thromb Vasc Biol* 1999, 19(12):2975-2980.**

**2. Schlitt A, Bickel C, Thumma P, Blankenberg S, Rupprecht HJ, Meyer J, Jiang XC: High plasma phospholipid transfer protein levels as a risk factor for coronary artery disease. *Arterioscler Thromb Vasc Biol* 2003, 23(10):1857-1862.**

**3. Riboli E, Hunt KJ, Slimani N, Ferrari P, Norat T, Fahey M, Charrondiere UR, Hemon B, Casagrande C, Vignat J *et al*: European Prospective Investigation into Cancer and Nutrition (EPIC): study populations and data collection. *Public health nutrition* 2002, 5(6B):1113-1124.**

**4. Panagiotakos DB, Pitsavos C, Chrysohoou C, Stefanadis C, Toutouzas P: Risk stratification of coronary heart disease in Greece: final results from the CARDIO2000 Epidemiological Study. *Preventive medicine* 2002, 35(6):548-556.**

**5. Sigurdsson E, Thorgeirsson G, Sigvaldason H, Sigfusson N: Prevalence of coronary heart disease in Icelandic men 1968-1986. The Reykjavik Study. *Eur Heart J* 1993, 14(5):584-591.**

**6. Latini V, Sole G, Doratiotto S, Poddie D, Memmi M, Varesi L, Vona G, Cao A, Ristaldi MS: Genetic isolates in Corsica (France): linkage disequilibrium extension analysis on the Xq13 region. *European journal of human genetics : EJHG* 2004, 12(8):613-619.**

**7. Screening experience and baseline characteristics in the West of Scotland Coronary Prevention Study. The WOSCOPS Study Group. West of Scotland Coronary Prevention Study. *Am J Cardiol* 1995, 76(7):485-491.**

**8. Keavney B, McKenzie C, Parish S, Palmer A, Clark S, Youngman L, Delepine M, Lathrop M, Peto R, Collins R: Large-scale test of hypothesised associations between the angiotensin-converting-enzyme insertion/deletion polymorphism and myocardial infarction in about 5000 cases and 6000 controls. International Studies of Infarct Survival (ISIS) Collaborators. *Lancet* 2000, 355(9202):434-442.**

**9. Hennekens CH, Buring JE, Manson JE, Stampfer M, Rosner B, Cook NR, Belanger C, LaMotte F, Gaziano JM, Ridker PM *et al*: Lack of effect of long-term supplementation with beta carotene on the incidence of malignant neoplasms and cardiovascular disease. *N Engl J Med* 1996, 334(18):1145-1149.**

**10. Mohrschladt MF, Westendorp RG, Gevers Leuven JA, Smelt AH: Cardiovascular disease and mortality in statin-treated patients with familial hypercholesterolemia. *Atherosclerosis* 2004, 172(2):329-335.**

**11. Drexel H, Aczel S, Marte T, Benzer W, Langer P, Moll W, Saely CH: Is atherosclerosis in diabetes and impaired fasting glucose driven by elevated LDL cholesterol or by decreased HDL cholesterol? *Diabetes Care* 2005, 28(1):101-107.**

**12. Marre M, Lievre M, Chatellier G, Mann JF, Passa P, Menard J: Effects of low dose ramipril on cardiovascular and renal outcomes in patients with type 2 diabetes and raised excretion of urinary albumin: randomised, double blind, placebo controlled trial (the DIABHYCAR study). *Bmj* 2004, 328(7438):495.**

**13. Sanghera DK, Demirci FY, Been L, Ortega L, Ralhan S, Wander GS, Mehra NK, Singh J, Aston CE, Mulvihill JJ *et al*: PPARG and ADIPOQ gene polymorphisms increase type 2 diabetes mellitus risk in Asian Indian Sikhs: Pro12Ala still remains as the strongest predictor. *Metabolism: clinical and experimental* 2010, 59(4):492-501.**

**14. Jukema JW, Bruschke AV, van Boven AJ, Reiber JH, Bal ET, Zwinderman AH, Jansen H, Boerma GJ, van Rappard FM, Lie KI *et al*: Effects of lipid lowering by pravastatin on progression and regression of coronary artery disease in symptomatic men with normal to moderately elevated serum cholesterol levels. The Regression Growth Evaluation Statin Study (REGRESS). *Circulation* 1995, 91(10):2528-2540.**

**15. Taylor GS, Muhlestein JB, Wagner GS, Bair TL, Li P, Anderson JL: Implementation of a computerized cardiovascular information system in a private hospital setting. *American heart journal* 1998, 136(5):792-803.**
